# Validation of Foot Placement Locations from Ankle Data of a Kinect v2 Sensor

**DOI:** 10.3390/s17102301

**Published:** 2017-10-10

**Authors:** Daphne Geerse, Bert Coolen, Detmar Kolijn, Melvyn Roerdink

**Affiliations:** 1 Department of Human Movement Sciences, Faculty of Behavioural and Movement Sciences, Vrije Universiteit Amsterdam, Amsterdam Movement Sciences, Van der Boechorststraat 7, 1081 BT Amsterdam, The Netherlands; h.coolen@vu.nl (B.C.); Detmar.Kolijn@rub.de (D.K.); m.roerdink@vu.nl (M.R.); 2 Department of Neurology, Leiden University Medical Center, Albinusdreef 2, 2333 ZA Leiden, The Netherlands

**Keywords:** Kinect v2 sensor, foot placement locations, gait, validation, orientation, TOST procedure

## Abstract

The Kinect v2 sensor may be a cheap and easy to use sensor to quantify gait in clinical settings, especially when applied in set-ups integrating multiple Kinect sensors to increase the measurement volume. Reliable estimates of foot placement locations are required to quantify spatial gait parameters. This study aimed to systematically evaluate the effects of distance from the sensor, side and step length on estimates of foot placement locations based on Kinect’s ankle body points. Subjects (n = 12) performed stepping trials at imposed foot placement locations distanced 2 m or 3 m from the Kinect sensor (distance), for left and right foot placement locations (side), and for five imposed step lengths. Body points’ time series of the lower extremities were recorded with a Kinect v2 sensor, placed frontoparallelly on the left side, and a gold-standard motion-registration system. Foot placement locations, step lengths, and stepping accuracies were compared between systems using repeated-measures ANOVAs, agreement statistics and two one-sided *t*-tests to test equivalence. For the right side at the 2 m distance from the sensor we found significant between-systems differences in foot placement locations and step lengths, and evidence for nonequivalence. This distance by side effect was likely caused by differences in body orientation relative to the Kinect sensor. It can be reduced by using Kinect’s higher-dimensional depth data to estimate foot placement locations directly from the foot’s point cloud and/or by using smaller inter-sensor distances in the case of a multi-Kinect v2 set-up to estimate foot placement locations at greater distances from the sensor.

## 1. Introduction

Quantitative gait assessments are a major undertaking in clinical settings (e.g., calibration procedures, patient-preparation time) and are costly due to expensive equipment [1]. The Microsoft Kinect v2 sensor may be a cheaper and easier to use alternative. It entails a RGB-D camera to create a depth image of its surrounding. Using machine-learning algorithms, the high-dimensional depth data can be reduced to 25 lower-dimensional three-dimensional (3D) body points of up to six people simultaneously, thereby eliminating the need for markers and calibration procedures [2]. The Kinect v2 sensor, originally developed for the gaming industry [2], has increasingly been studied in terms of its usability for quantitative gait assessments [3,4,5,6,7,8,9,10]. These studies collectively revealed that the Kinect v2 sensor is a promising tool for measuring spatiotemporal gait parameters [3,4,5,6,7,8,9,10].

**Figure 1:**
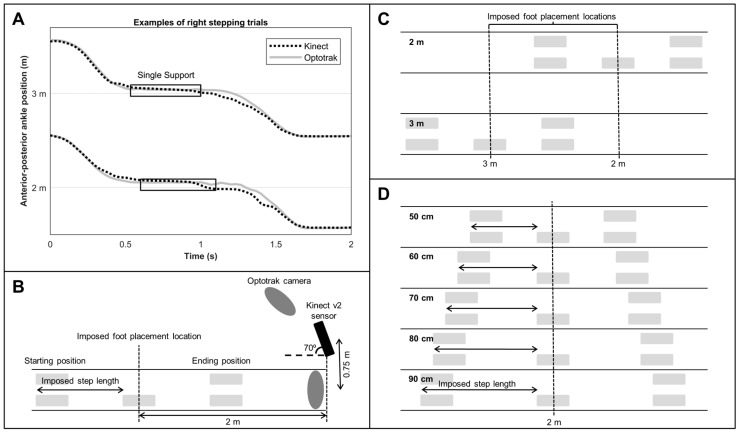
(**A**) Representative example of the right anterior-posterior ankle position for the Kinect v2 sensor (dotted black line) and a gold-standard Optotrak system (solid gray line) during two right stepping trials (at 2 m and 3 m distance from the sensor with the Kinect v2 sensor positioned at 0 m and walking direction towards the sensor). The single-support phase is indicated by the black boxes; (**B**) Schematic overview of the experimental set-up together with a right stepping trial at a 2 m distance from the sensor; (**C**) Schematic overview of the two imposed foot placement locations distanced 2 m (top) and 3 m (bottom) from the Kinect v2 sensor for right stepping trials; and, (**D**) Schematic overview of the different imposed step lengths for right stepping trials at a 2 m distance from the sensor.

Spatial gait parameters, such as step length, are quantified from estimates of foot placement locations, which are approximated from 3D positional data of Kinect’s ankle body points [3,6,7,8,9]. However, Kinect’s estimate of the ankle position seems to gradually change during the gait cycle in the anterior-posterior direction when compared to a gold standard, a phenomena that we observed in our own studies [6,7] as well as in other studies [9,11]. The influence of this gradual change in the anterior-posterior ankle position, as depicted in Figure 1A, on approximated foot placement locations has never been systematically examined, which seems essential given that yet unknown effects of distance from the Kinect v2 sensor, side and step length may affect outcome measures of quantitative gait assessments.

The objective of this study is to systematically compare foot placement locations, as approximated from ankle body point data, and associated estimates of step length and stepping accuracy between the Kinect v2 sensor and a gold-standard motion-registration system. To this end, the effect of distance to the Kinect v2 sensor, left and right foot placement locations (side) and imposed step lengths will be examined. We expect that foot placement locations, step lengths, and stepping accuracies will agree well between systems, without systematic between-systems effects of distance, side and imposed step length.

## 2. Methods

### 2.1. Subjects

A group of 12 healthy subjects (mean [range]: age 28 [21 43] years, height 177 [158 190] cm, weight 74 [56 95] kg, 6 males) participated in this experiment. The Ethics Committee of the Department of Human Movement Sciences of the Vrije Universiteit Amsterdam (Amsterdam, The Netherlands) approved the study (ECB 2015-55). All of the subjects gave written informed consent prior to participation.

### 2.2. Experimental Set-Up and Procedure

Body points’ time series of the lower extremities were recorded with a Kinect v2 sensor and a gold-standard Optotrak system (Northern Digital Inc., Waterloo, ON, Canada). For the current study, the orientation and position of the Kinect sensor was in agreement with those of the Kinect sensors of a validated multi-Kinect v2 set-up for gait assessments (i.e., an angle of 70 degrees relative to the movement direction and a perpendicular distance of 0.75 m to the center of the area of interest; [6,7]; Figure 1B). Multiple Kinect v2 sensors placed in a frontoparallel orientation (70 degrees) alongside a walkway allows for a larger measurement volume for quantitative gait assessments [6,7,9]. Two Optotrak cameras were needed to cover the same area as the Kinect sensor (see Figure 1B for a schematic overview). A spatial calibration grid was used to spatially align the coordinate systems of the two motion-registration systems to a common coordinate system, as detailed in [7].

As in [6,7], the Kinect for Windows Software Development Kit (SDK 2.0, www.microsoft.com) was used to obtain the 3D time series of 25 body points by means of inbuilt and externally validated human-pose estimation algorithms [3,6,7,8,9,12,13,14]. Kinect data were sampled at 30 Hz using custom-written software utilizing the SDK 2.0. For the Optotrak system, Smart Marker Rigid Bodies (Northern Digital Inc., Waterloo, ON, Canada) were attached to the body segments of the lower extremities (lower abdomen, upper legs, and lower legs) and virtual markers were assigned to these rigid bodies using a 3-marker digitizing probe using First Principles data acquisition software (see Supplementary Material S1). The positions of the virtual markers were 14 anatomical landmarks chosen to match the body points of the Optotrak system with the body points of the lower body of the Kinect system (see Supplementary Material S1). The positions of these virtual markers were averaged in all directions for each sample to obtain the positions of seven matched body points (see Supplementary Material S1). Optotrak data were sampled at 60 Hz.

Subjects performed multiple stepping trials with foot placement locations being guided by five shoe-size-matched stepping stones (Figure 1B) presented using a projector (Vivitek D7180HD, ultra-short-throw Full HD projector), which was spatially aligned to the common coordinate system of the two motion-registration systems. The center of the middle stepping stone was positioned at two different imposed foot placement locations, distanced at either 2 m or 3 m from the Kinect sensor (Figure 1C). These distances ensure a high resolution of the depth data [15], and thus minimize the influence of depth resolution on the outcome measures. The middle stepping stone was either projected for the left or right foot depending on its mediolateral position. The position of the stepping stones indicating the starting and ending positions were determined based on the imposed step lengths (50 cm, 60 cm, 70 cm, 80 cm, or 90 cm; Figure 1D). Step width was set at 20 cm to ensure that the stepping stones did not overlap. Subjects were asked to stand as accurately as possible in the stepping stones indicating the starting position and then step with their left or right foot (depending on the imposed stepping pattern) in the middle stepping stone and end with both feet in the stepping stones indicating the ending position, thereby making a stepping movement. All of the trials were performed twice, yielding a total of 40 trials (i.e., at 2 m and 3 m distances, with the left and right side, at five imposed step lengths for two repetitions). Trials were block-randomized for distance and side.

### 2.3. Data Pre-Processing and Analysis

Data pre-processing followed established procedures [6,7] using Matlab R2015a (The MathWorks Inc., Natick, MA, USA). Body points of the Kinect system classified as inferred (i.e., when Kinect’s human-pose estimation software can only indirectly derive the position of the body point due to partial occlusion for instance) were removed from the time series. Body point’s time series were linearly interpolated to ensure a constant sampling frequency of 30 Hz, without filling in the missing data points. Data points were removed from the time series when they did not meet our criteria for valid human pose estimation (e.g., a minimum of 15 out of the 25 possible body points should be labeled as tracked, including the head and at least one foot and ankle, without outliers in segment lengths). Optotrak body point’s time series were down-sampled to 30 Hz. These data are available as Supplementary Material (Supplementary Material S2). Body point’s time series of the spine base and left and right ankle in the anterior-posterior direction were interpolated with a spline algorithm and were used for the calculation of the outcome measures. Percentages of missing data for these body points’ time series were on average 3.9% for the Kinect system and 0.6% for the Optotrak system, with maximum percentages of missing data of 21.4% and 20.1%, respectively.

The outcome measures were foot placement location, step length, and stepping accuracy. Foot placement locations were estimated from the anterior-posterior ankle position during the single-support phase (i.e., between foot off and foot contact of the contralateral foot; estimates of foot off and foot contact were defined as the minima and maxima of the anterior-posterior time series of the ankle relative to that of the spine base; [6,7,16]). Foot placement locations were transformed to center of the foot, using the ankle positions of the feet aligned with the stepping stones of the starting positions as a reference. To this end, the average distance of the left and right ankle to the center of the stepping stones was calculated over the episode of five samples before step initiation with the lowest amount of variation for each trial. Subsequently, foot placement locations were normalized to imposed foot placement locations (i.e., imposed foot placement location was subtracted from the measured foot placement location to correct for arbitrary effects in foot placement location as a function of the two imposed distances from the sensor). Step length was defined as the anterior-posterior distance between the starting position and the (non-normalized) foot placement location (see arrows in Figure 1D). Stepping accuracy was defined as the standard deviation over the signed normalized foot placement locations over step lengths and repetitions and was calculated per system, distance, and side.

### 2.4. Statistical Analysis

One trial was accidentally not recorded with the Kinect system (experimenter forgot to start the recording without noticing it), resulting in missing data for foot placement location and step length for one participant (3 m distance, right side, 80 cm and repetition #2). Since missing data in a repeated-measures ANOVA will lead to the entire removal of that participant from the analysis, we decided to use this single observation for this participant and to average over the two repetitions for all other conditions and participants, yielding a single value for each combination of system, distance, side, and imposed step length for all of the participants. Two participants had to be excluded from further analyses due to displaced cluster markers of the Optotrak system.

All outcome measures (foot placement location, step length, and stepping accuracy) were compared between systems using repeated-measures ANOVAs (IBM SPSS Statistics 24). For foot placement locations, and step lengths, a System (Kinect, Optotrak) by Distance (2 m, 3 m) by Side (left, right foot placement locations) by Imposed step length (50 cm, 60 cm, 70 cm, 80 cm, 90 cm) repeated-measures ANOVA was conducted. For stepping accuracy, a System by Distance by Side repeated-measures ANOVA was conducted. The assumption of sphericity was verified according to Girden [17]. The Huynh-Feldt correction was applied if the Greenhouse-Geisser’s epsilon exceeded 0.75; otherwise, the Greenhouse-Geisser correction was used. The main effects were examined with a Least Significant Difference post-hoc test for factors with two levels and contrast analyses for factors with more than two levels. Paired-samples *t*-tests were used for significant interactions involving the factor System, focusing on between-systems comparisons. Effect sizes were quantified with *η_p_^2^*.

In addition to the ANOVAs testing between-systems differences, we also performed agreement statistics to examine the agreement between the systems. The between-systems agreement was determined using intraclass correlation for absolute agreement (ICC_(A,1)_) and consistency (ICC_(C,1)_; [18]) using Matlab R2015a, with values above 0.60 and 0.75, representing good and excellent agreement, respectively [19]. Both types of ICCs were used in order to determine the influence of a potential systematic between-systems bias in the agreement. The ICCs were complemented by mean differences and precision values obtained with a Bland–Altman analysis (i.e., the bias [Kinect-Optotrak] and the limits of agreement [LoA], respectively; [20]).

In view of the low between-subject variation due to the imposed foot placement locations and step lengths, which may hinder the reliability of the ICCs [21], the outcome measures were also analyzed for between-systems equivalence using two one-sided *t*-tests (TOST; utilizing the TOSTER module in jamovi 0.7.3.2; [22]). For this analysis, the 90% confidence interval of the between-systems difference should be within pre-determined equivalence bounds for which the systems can be deemed equivalent. These bounds were conservatively set based on the LoA intervals found in [7]. That is, for foot placement locations and step lengths, the equivalence bounds were set at ±2.145 cm (i.e., the smallest LoA interval of the obstacle-avoidance margins, which were similarly based on estimates of a single foot placement location; [7]). For stepping accuracies, the smallest LoA interval was used of the stepping accuracies obtained for precision-stepping trials to a sequence of regularly spaced stepping stones with imposed step lengths of 50 cm, 60 cm, 70 cm, 80 cm, and 90 cm ([7]; same step lengths as in the current study), resulting in equivalence bounds of ±0.685 cm.

## 3. Results

Table 1 shows the data of all outcome measures together with the agreement statistics (bias, 95% LoA, ICC_(A,1)_ and ICC_(C,1)_) and TOST statistics.

### 3.1. Foot Placement Locations

**Figure 2:**
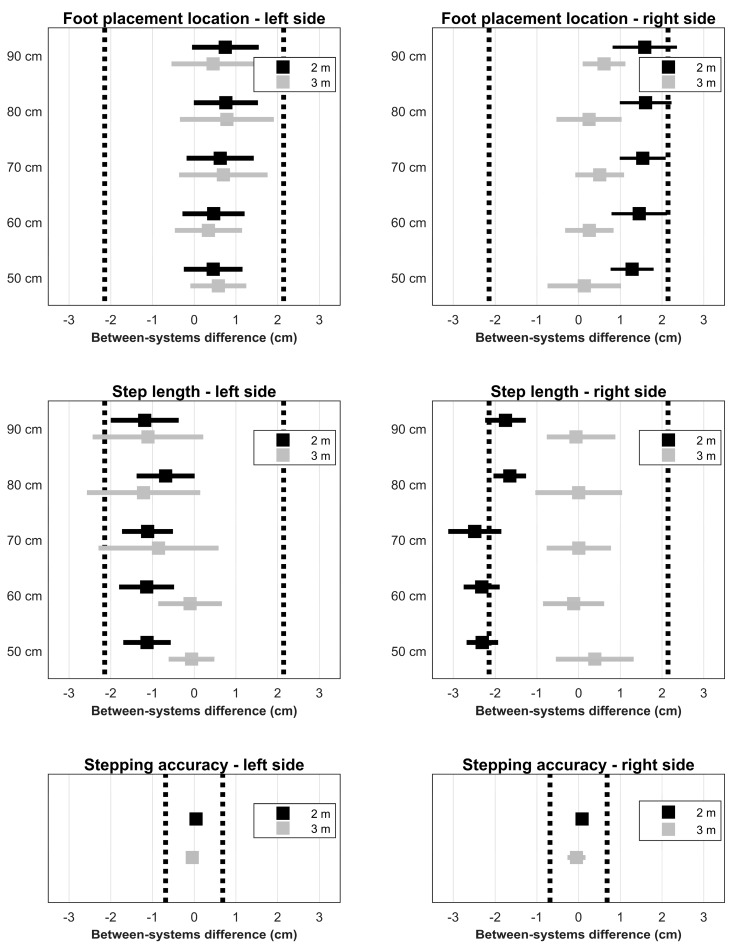
Results of the two one-sided *t*-tests, showing the between-systems differences and the 90% confidence intervals of all conditions for foot placement location, step length, and stepping accuracy.

A significant main effect of System (*F*(1,9) = 5.87, *p* = 0.038, *η_p_^2^* = 0.395) was found on foot placement locations. Kinect estimated foot placement locations 0.76 cm posterior as compared to the Optotrak system. No other main or interaction effects were found, although there was a trend towards significant System×Imposed step length (*F*(2.6,23.4) = 2.83, *p* = 0.067, *η_p_^2^* = 0.239) and System×Distance×Side (*F*(1,9) = 4.66, *p* = 0.059, *η_p_^2^* = 0.341) interactions. There seemed to be a larger between-systems difference for the right foot placement location at 2 m when compared to the other conditions (see top panels in Figure 2). Regarding the equivalence tests, right foot placement locations at 2 m were found to be nonequivalent for 80 cm (*p* = 0.072) and 90 cm (*p* = 0.110), while all other foot placement locations were found to be equivalent (*p* < 0.045). Note that in some cases the systems can be considered equivalent, as their 90% confidence intervals do not cross the equivalence bounds (i.e., no meaningful effect), and at the same time be statistically different in a *t*-test because the confidence intervals of the between-systems differences do not include zero (e.g., right foot placement locations at the 2 m distance for imposed step lengths of 50 cm, 60 cm, and 70 cm; Table 1, Figure 2).

### 3.2. Step Length

A main effect of System was found on step length (*F*(1,9) = 12.24, *p* = 0.007, *η_p_^2^* = 0.576). On average, Kinect underestimated step length with 0.94 cm as compared to the Optotrak system, a finding in line with abovementioned between-systems difference in foot placement locations. There was also a very strong effect of imposed step length on performed step length (*F*(2.8,25.0) = 8167.28, *p* < 0.001, *η_p_^2^* = 0.999; with significant linear [*F*(1,9) = 23285.32, *p* < 0.001, *η_p_^2^* = 1.000] and quadratic [*F*(1,9) = 11.73, *p* = 0.008, *η_p_^2^* = 0.566] contrasts); step lengths increased with increasing imposed step lengths.

**Figure 3:**
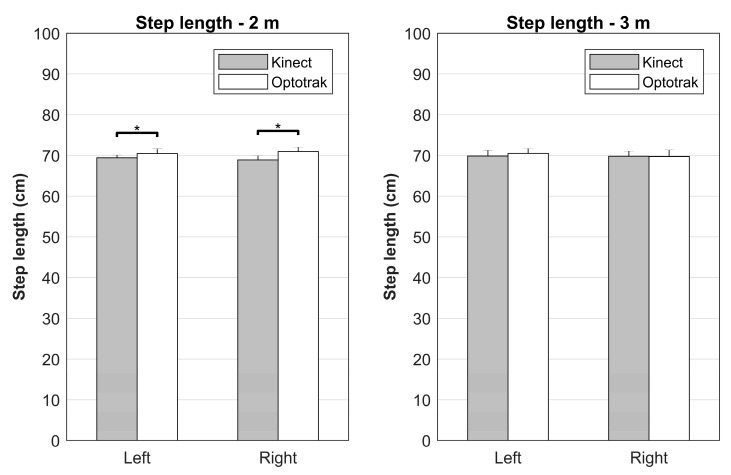
Visual representation of the interaction effect of System, Distance, and Side. The significant between-systems bias in step length was only found at the 2 m distance (indicated by the asterisks) and more strongly so for right step lengths (indicated by the significantly larger between-systems difference for the right step length).

**Figure 4:**
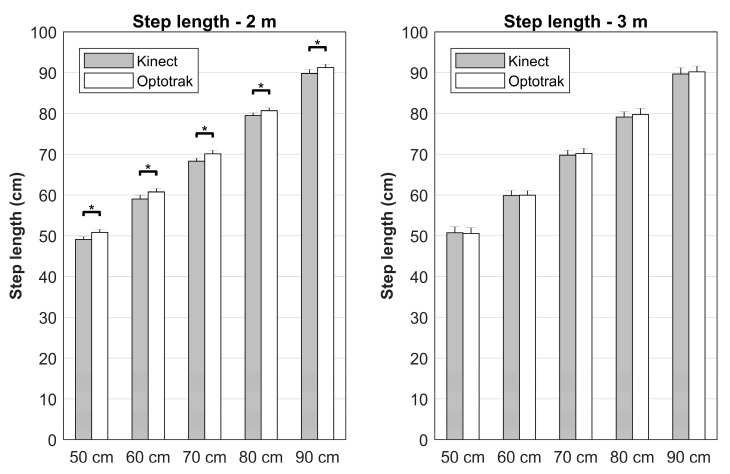
Visual representation of the interaction effect of System, Distance and Imposed step length. Significant between-systems differences in step length were only found at 2 m, with larger biases for larger imposed step lengths.

Furthermore, significant System×Distance (*F*(1,9) = 13.12, *p* = 0.006, *η_p_^2^* = 0.593) and System×Distance×Side (*F*(1,9) = 12.26, *p* = 0.007, *η_p_^2^* = 0.577) interactions were observed. The significant between-systems bias was only found at the 2 m distance and more strongly so for right step lengths (Figure 3), indicated by the significantly larger between-systems difference for the right step length at 2 m (*t*(9) = 3.51, *p* = 0.007). In addition, Distance×Imposed step length (*F*(4,36) = 5.45, *p* = 0.002, *η_p_^2^* = 0.377; with significant linear by linear [*F*(1,9) = 18.31, *p* = 0.002, *η_p_^2^* = 0.670] and linear by fourth order [*F*(1,9) = 13.35, *p* = 0.005, *η_p_^2^* = 0.597] contrasts) and System×DistancexImposed step length (*F*(2.8,25.1) = 4.35, *p* = 0.015, *η_p_^2^* = 0.326) interactions were found; significant between-systems differences were again only found at the 2 m distance, with the smallest between-systems bias for 80 cm (Table 1, Figure 4).

Step lengths were generally found to be equivalent (most *p* < 0.030) with some exceptions for the right step length at 2 m, in agreement with the System×Distance×Imposed step length interaction, and the left step length at 3 m due to a relatively large between-subject variation (Figure 2).

### 3.3. Stepping Accuracy

For stepping accuracy, no significant main or interaction effects were found (all *p* > 0.089, all *η_p_^2^* < 0.287). There was a trend towards significance for the System×Distance (*F*(1,9) = 3.62, *p* = 0.089, *η_p_^2^* = 0.287) interaction. Kinect seemed to slightly underestimate stepping accuracy at the 2 m distance, and to slightly overestimate stepping accuracy at the 3 m distance (i.e., see the non-significant positive and negative biases in Table 1, respectively). Nevertheless, stepping accuracy was found to be equivalent between the systems (*p* < 0.001; Figure 2).

## 4. Discussion

The objective of this study was to systematically compare foot placement locations, as approximated from ankle body point data, and associated estimates of step length and stepping accuracy between the Kinect v2 sensor and a gold-standard Optotrak system. We expected that foot placement locations, step lengths, and stepping accuracies all agreed well between systems, without systematic between-systems effects of distance from the sensor, side and imposed step length. However, our results revealed a small but significant between-systems difference in foot placement locations and step lengths; Kinect estimated foot placement locations on average 0.76 cm posterior and consequently underestimated step length by 0.94 cm when compared to the Optotrak system. Note that these biases were predominantly found for the 2 m distance and were more pronounced for the right side. Nevertheless, stepping accuracies and estimates of foot placement locations and step lengths were generally statistically equivalent (i.e., no statistically meaningful between-systems bias, as evidenced by a statistically significant TOST), with a few nonequivalent exceptions in foot placement locations and step lengths mostly for the right side at the 2 m distance (Table 1, Figure 2).

Two factors may have mediated the larger between-systems differences for the right side at the 2 m distance: (1) depth occlusion and (2) body orientation relative to the Kinect sensor. Since the Kinect sensor was positioned frontoparallelly on the left side of the participant, the right leg could be partially occluded by the swinging left leg during the stepping movement, and more strongly so nearby the sensor, which may have affected the outcomes. In the Supplementary Material (Supplementary Material S3) we describe an additional analysis aimed at examining the role of occlusion (and associated interpolation of the missing data) as a factor mediating the larger between-systems differences found for the foot placement locations of the right side at the 2 m distance. Based on the results we can conclude that depth occlusion did not cause the larger between-systems bias.

**Figure 5:**
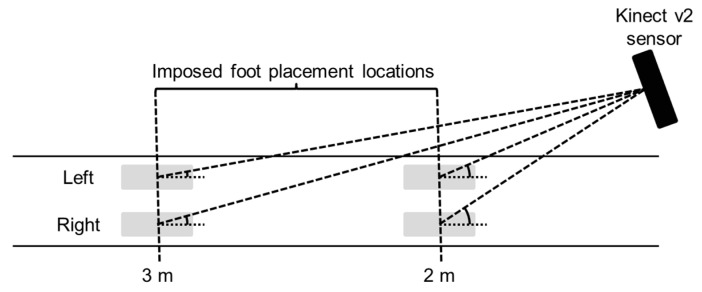
An overview of the influence of distance from the sensor and body side on body orientation relative to the Kinect sensor.

Could the second factor, body orientation relative to the Kinect sensor, then explain the between-systems differences for the right side at 2 m distance from the sensor? As can be seen in Figure 5, the orientation relative to the Kinect sensor changes with distance from the sensor and body side: from quite frontally for the left side at the 3 m distance to a more frontoparallel orientation for the right side at the 2 m distance. Orientation relative to the sensor likely affects the depth image of shank and foot segments due to orientation-based differences in self-occlusion of those body segments, which might influence the estimation of the position of the ankles from the point clouds by the machine-learning algorithm (cf. Figure 5B in [9]), and as such estimates of foot placement locations. Indeed, Wang et al. [23] showed that the positional error in body point estimates increases with deviations from a frontal orientation relative to the Kinect v2 sensor, especially so for body points of the body side that was turned away from the sensor. The turned-away body side was the right side in the current study, with the greatest deviations from a frontal orientation at the 2 m distance. This was also the condition with a meaningful between-systems bias in estimated foot placement locations, making body orientation relative to the sensor a very likely cause for the observed between-systems differences.

Knowing that body orientation relative to the sensor affects body point estimation, we will now discuss ways to minimize orientation biases in (multi-)Kinect set-ups for measuring gait with (a) sensor(s) placed alongside a walkway. A first recommendation could be to use sensors on both sides of a walkway in order to average out side-dependent orientation biases. Müller et al. [9] recently compared one-sided and two-sided multi-Kinect v2 set-ups to a gold-standard motion-registration system. They found superior between-systems agreement in step widths for the two-sided set-up, suggesting that mediolateral orientation biases, which are opposite in direction for the two sides, can indeed be successfully averaged out. Unfortunately, a two-sided set-up will not help to solve anterior-posterior orientation biases because these biases are similar in direction for both sides, with greater biases closer to the sensor. A second recommendation could be to use Kinect’s higher-dimensional depth data to estimate foot placement locations directly from the foot’s point cloud instead of approximating it from the lower-dimensional ankle body points’ time series. Point clouds are robust, richer in information, and are likely less prone to orientation errors. Previous studies indeed found superior results for outcome measures (i.e., stride durations, stride lengths, and step asymmetries) derived from Kinect’s higher-dimensional point clouds than for their counterparts derived from Kinect’s lower-dimensional body points’ time series [24,25,26]. As point clouds contain more information about the foot, they may additionally allow for finer-grained foot-related gait parameters, which seem particularly useful in clinical populations with gait deviations and foot deformations. Although point clouds may thus be a very useful alternative for determining foot placement locations, the higher dimensionality of the point clouds place greater demands on data handling. This is not much of a concern for post-processing, but will be a burden for real-time processing of gait data from multiple Kinect sensors for gait-dependent event control (e.g., suddenly projecting an obstacle at the location where one will step next; [7]). A more parsimonious solution, therefore, seems to be to collect body point data at greater distances from the sensor, for which we have shown that they are less prone to orientation biases. In the case of a multi-Kinect v2 set-up, this implies smaller inter-sensor distances to create more overlap between the measurement volumes of the sensors. Consequently, body point data nearby the sensor, which suffers from orientation biases, can be ignored because the same body points are already detected by the more distant sensor whose data is minimally affected by orientation biases.

A limitation of this study was that the effect of distance to the sensor was assessed in a rather coarse-grained manner (i.e., 2 levels, at 2 m and 3 m from the sensor). As a consequence, the precise cut-off for ignoring nearby data to circumvent orientation biases remains unknown. Another limitation is that two participants had to be excluded due to displaced cluster markers of the Optotrak system during the experiment, resulting in a relatively small sample size. The sample consisted of healthy adults without gait deviations, whose gait may not be representative for the gait of various patient groups. Nevertheless, there is no reason to expect inferior depth images or body point estimation of the lower extremities for persons with gait deviations [4], so the same recommendations apply for negating orientation biases when the Kinect v2 sensor is used for quantitative gait assessments in clinical populations.

## 5. Conclusions

There is a meaningful between-systems difference in foot placement locations, albeit only nearby the sensor and exclusively for the body side turned away from the sensor (in our study the right side at a 2 m distance). This distance by side between-systems effect is not mediated by depth occlusion through the contralateral swinging leg, but is likely caused by body orientation differences relative to the sensor. Such orientation effects might be reduced by using the higher-dimensional depth data to estimate foot placement locations directly from the foot’s point cloud and/or by using smaller inter-sensor distances in the case of a multi-Kinect v2 set-up, allowing for foot placement estimations at greater distances from the sensor.

## Figures and Tables

**Table 1:** Mean values, between-subjects standard deviations (SD), agreement statistics (bias, limits of agreement [95% LoA] and intraclass correlation coefficients for absolute agreement [ICC_(A,1)_] and consistency [ICC_(C,1)_]) and results of the two one-sided *t*-tests (TOST) for the foot placement locations, step lengths and stepping accuracies for all combinations of the independent variables Distance (2 m, 3 m), Side (left, right) and Imposed Step Length (50 cm, 60 cm, 70 cm, 80 cm, 90 cm).

				Kinect v2	Optotrak				TOST
				Mean ± SD	Mean ± SD	Bias [95% LoA]	ICC_(A,1)_	ICC_(C,1)_	*p*-Value
**Foot placement location (cm)**	**2 m**	**Left**	**50 cm**	−0.02 ± 0.87	−0.48 ± 1.33	0.46 [−1.93 2.85]	0.403	0.413	<0.001
			**60 cm**	−0.04 ± 1.24	−0.51 ± 1.67	0.47 [−2.06 3.00]	0.608	0.615	<0.001
			**70 cm**	0.02 ± 0.84	−0.61 ± 1.30	0.62 [−2.10 3.35]	0.178	0.193	0.004
			**80 cm**	0.47 ± 0.96	−0.29 ± 1.10	0.76 [−1.84 3.36]	0.147	0.174	0.005
			**90 cm**	0.01 ± 1.06	−0.73 ± 1.03	0.75 [−1.95 3.45]	0.115	0.135	0.005
		**Right**	**50 cm**	0.34 ± 1.35	−0.95 ± 1.03	1.28 * [−0.46 3.03]	0.471	0.727	0.007
			**60 cm**	0.68 ± 1.77	−0.77 ± 1.18	1.45 * [−0.79 3.70]	0.493	0.709	0.045
			**70 cm**	1.32 ± 1.17	−0.22 ± 0.79	1.54 * [−0.33 3.41]	0.254	0.544	0.038
			**80 cm**	0.57 ± 1.21	−1.04 ± 0.68	1.61 * [−0.48 3.69]	0.182	0.414	0.072 **
			**90 cm**	0.65 ± 1.52	−0.94 ± 1.04	1.59* [−1.02 4.20]	0.282	0.478	0.110 **
	**3 m**	**Left**	**50 cm**	−0.22 ± 1.16	−0.80 ± 1.13	0.58 [−1.69 2.85]	0.458	0.493	0.001
			**60 cm**	−0.10 ± 1.09	−0.44 ± 1.30	0.34 [−2.38 3.07]	0.334	0.325	0.001
			**70 cm**	−0.55 ± 1.57	−1.25 ± 1.50	0.70 [−2.89 4.29]	0.275	0.284	0.017
			**80 cm**	−0.00 ± 1.96	−0.79 ± 1.91	0.79 [−3.02 4.60]	0.480	0.495	0.027
			**90 cm**	−0.62 ± 1.51	−1.07 ± 1.82	0.45 [−2.92 3.83]	0.477	0.469	0.006
		**Right**	**50 cm**	−0.49 ± 1.48	−0.63 ± 1.64	0.14 [−2.84 3.11]	0.550	0.526	0.001
			**60 cm**	0.28 ± 1.59	0.02 ± 1.85	0.26 [−1.71 2.22]	0.836	0.831	<0.001
			**70 cm**	0.54 ± 1.34	0.04 ± 1.62	0.51 [−1.47 2.48]	0.744	0.770	<0.001
			**80 cm**	0.25 ± 1.42	−0.00 ± 2.13	0.25 [−2.39 2.89]	0.736	0.723	<0.001
			**90 cm**	0.04 ± 1.32	−0.57 ± 1.33	0.61 [−1.13 2.35]	0.717	0.777	<0.001
**Step length (cm)**	**2 m**	**Left**	**50 cm**	49.19 ± 1.38	50.32 ± 1.46	−1.13 * [−3.05 0.80]	0.590	0.761	0.005
			**60 cm**	59.32 ± 1.41	60.46 ± 1.50	−1.14 * [−3.36 1.08]	0.546	0.696	0.010
			**70 cm**	69.09 ± 0.94	70.20 ± 1.27	−1.12 * [−3.18 0.95]	0.380	0.554	0.006
			**80 cm**	79.45 ± 1.09	80.13 ± 1.40	−0.68 [−3.04 1.67]	0.492	0.542	0.002
			**90 cm**	90.06 ± 0.98	91.25 ± 1.38	−1.19 * [−3.95 1.58]	0.213	0.304	0.030
		**Right**	**50 cm**	49.02 ± 1.73	51.33 ± 1.37	−2.31 * [−3.59 −1.03]	0.439	0.913	0.773 **
			**60 cm**	58.73 ± 1.44	61.05 ± 1.30	−2.32 * [−3.77 −0.87]	0.353	0.854	0.762 **
			**70 cm**	67.50 ± 1.66	69.98 ± 1.39	−2.49 * [−4.64 −0.33]	0.325	0.744	0.825 **
			**80 cm**	79.52 ± 1.03	81.17 ± 1.25	−1.65 * [−2.97 −0.32]	0.411	0.827	0.022
			**90 cm**	89.49 ± 1.61	91.24 ± 1.72	−1.75 * [−3.41 −0.10]	0.566	0.871	0.089 **
	**3 m**	**Left**	**50 cm**	50.66 ± 1.35	50.73 ± 1.16	−0.06 [−1.92 1.80]	0.737	0.717	<0.001
			**60 cm**	60.18 ± 1.50	60.28 ± 1.38	−0.10 [−2.69 2.49]	0.605	0.581	<0.001
			**70 cm**	69.92 ± 1.91	70.78 ± 1.82	−0.85 [−5.72 4.02]	0.110	0.112	0.067 **
			**80 cm**	78.81 ± 2.14	80.02 ± 1.78	−1.21 [−5.81 3.38]	0.258	0.289	0.120 **
			**90 cm**	89.64 ± 1.99	90.74 ± 1.78	−1.11 [−5.60 3.38]	0.242	0.266	0.093 **
		**Right**	**50 cm**	50.79 ± 2.00	50.41 ± 2.10	0.39 [−2.77 3.55]	0.699	0.690	0.004
			**60 cm**	59.53 ± 1.97	59.65 ± 2.15	−0.12 [−2.60 2.36]	0.826	0.812	<0.001
			**70 cm**	69.60 ± 1.22	69.59 ± 1.54	0.01 [−2.61 2.62]	0.568	0.542	<0.001
			**80 cm**	79.44 ± 1.43	79.43 ± 2.29	0.00 [−3.52 3.53]	0.582	0.556	0.002
			**90 cm**	89.69 ± 1.32	89.63 ± 1.78	0.06 [−2.72 2.84]	0.615	0.590	<0.001
**Stepping accuracy (cm)**	**2 m**	**Left**		1.33 ± 0.29	1.28 ± 0.33	0.05 [−0.24 0.33]	0.892	0.892	<0.001
		**Right**		1.30 ± 0.30	1.21 ± 0.26	0.08 [−0.18 0.35]	0.855	0.884	<0.001
	**3 m**	**Left**		1.52 ± 0.54	1.57 ± 0.61	−0.05 [−0.51 0.41]	0.922	0.917	<0.001
		**Right**		1.43 ± 0.47	1.48 ± 0.54	−0.05 [−0.78 0.68]	0.745	0.729	<0.001

* Significant between-systems difference (*p* < 0.05); ** Non-significant two one-sided *t*-tests, indicating nonequivalence (TOST *p* > 0.05).

## Supplementary Materials

The following are available online at http://www.mdpi.com/1424-8220/17/10/2301/s1, Supplementary Material S1: Marker set-up of the Optotrak system, Supplementary Material S2: Body points’ time series in the anterior-posterior, mediolateral and vertical direction for the Kinect v2 sensor and the Optotrak system, Supplementary Material S3: Examination of the effect of occlusion in Kinect v2 ankle data.
