# Supplementary material for: Validation of Foot Placement Locations from Ankle Data of a Kinect v2 Sensor
Source: Sensors (Basel). 2017 Oct 10;17(10):2301. doi: 10.3390/s17102301 (PMC5677405; doi:10.3390/s17102301)
Supplement: Supplementary file 1 [file sensors-17-02301-s001.zip › Supplementary Material/Supplementary Material S1.pdf]

Marker set-up of the Optotrak system

Supplementary material to: Daphne Geerse, Bert Coolen, Detmar Kolijn and Melvyn Roerdink. Validation of foot placement locations from ankle data of a Kinect v2 sensor. Sensors.

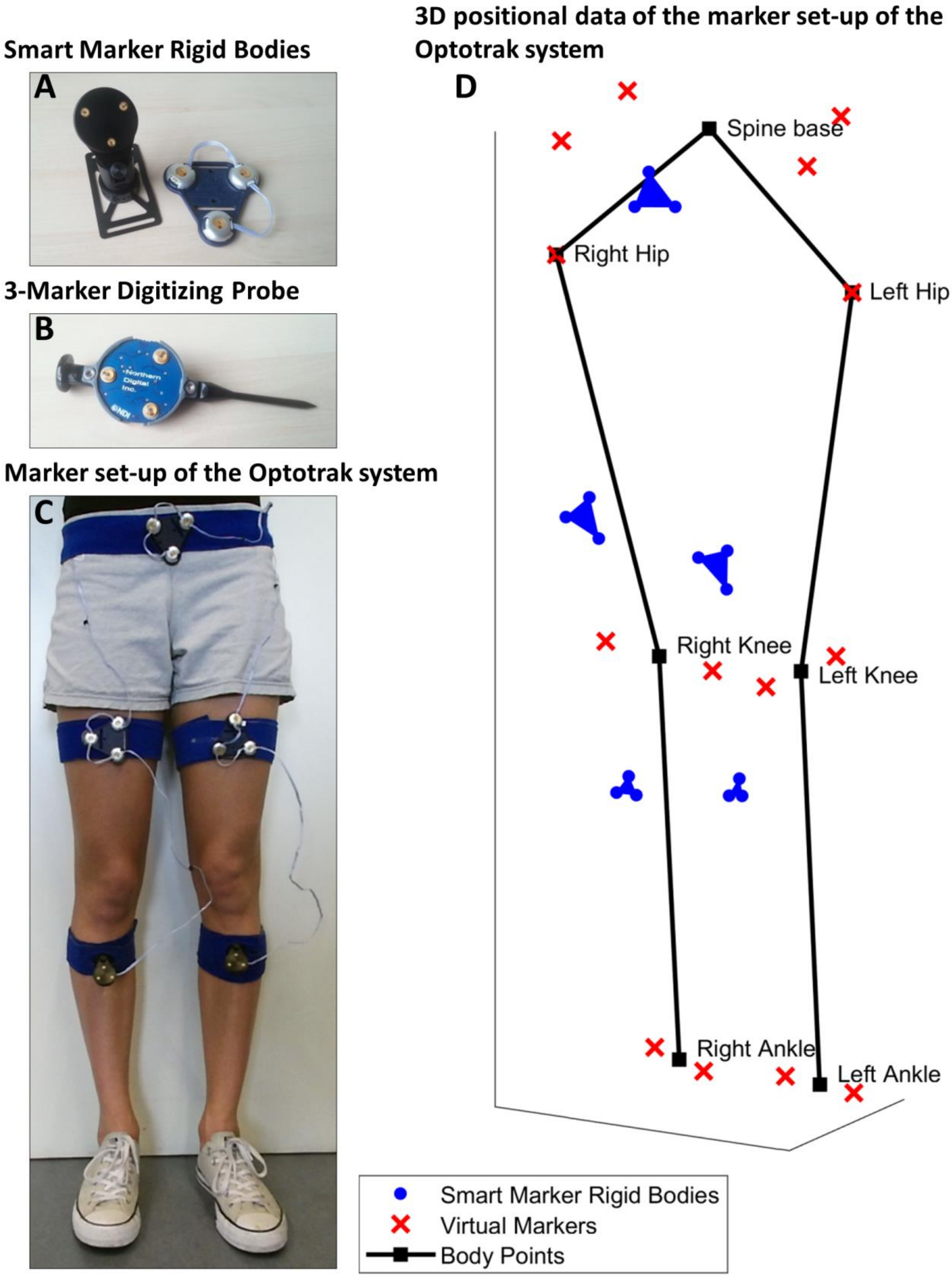

Figure 1. A) Smart Marker Rigid Bodies of the Optotrak system. B) 3-marker digitizing probe for assigning virtual markers to the Smart Marker Rigid Bodies. C) Overview of the marker set-up of the Optotrak system. D) Schematic overview of the 3D positional data of the marker set-up of the Optotrak system. Smart Marker Rigid Bodies (presented in blue) were attached to the body segments of the

lower abdomen, upper legs, and lower legs. Virtual markers (red crosses) were assigned to these rigid bodies using a 3-marker digitizing probe. The positions of the virtual markers were 14 anatomical landmarks chosen to match the body points of the Optotrak system with the body points of the Kinect system (Table 1). The positions of these virtual markers were averaged in all directions for each sample to obtain the positions of seven matched body points (Table 1; black squares).

Table 1. Overview of Optotrak marker data for deriving body points resembling Kinect body points.

Kinect body points	Smart Marker Rigid Body position	Virtual marker position
**Spine base**	Lower abdomen	Right and left anterior superior and posterior superior iliac spine
**Hips**	Upper legs	Trochantor major
**Knees**	Upper legs	Medial and lateral condyles
**Ankles**	Lower legs	Medial and lateral malleoli
