# Supplementary material for: Validation of Foot Placement Locations from Ankle Data of a Kinect v2 Sensor
Source: Sensors (Basel). 2017 Oct 10;17(10):2301. doi: 10.3390/s17102301 (PMC5677405; doi:10.3390/s17102301)
Supplement: Supplementary file 1 [file sensors-17-02301-s001.zip › Supplementary Material/Supplementary Material S3.pdf]

Examination of the effect of occlusion in Kinect v2 ankle data

Supplementary material to: Daphne Geerse, Bert Coolen, Detmar Kolijn and Melvyn Roerdink. Validation of foot placement locations from ankle data of a Kinect v2 sensor. Sensors.

In this supplementary material we describe an additional analysis aimed at examining the role occlusion (and the associated interpolation of missing data) may have played in the larger between-systems differences observed for the right side at the 2m distance. First, we compared the amount of occlusion in the Kinect v2 data between distances and sides during the single-support phase. Second, we introduced occlusion (i.e., based on observed occlusion for the right side) to the data of the typically unoccluded left side to examine its effect on estimates of foot placement locations. If these foot placement locations are systematically affected by the introduced occlusion at the 2m distance only, occlusion (and the associated interpolation of missing data) likely caused the observed between-systems differences for the 2m distance for right foot placements.

Methods

Data analysis

The first step in the analysis was to compare the amount of occlusion (i.e., missing data) in the Kinect v2 data between distances and sides. Therefore, raw Kinect v2 body point's time series of the ankles without interpolation of the missing data points were used. The amount of occlusion was determined during the single-support phase (i.e., between foot off and foot contact of the contralateral foot), since foot placement locations were estimated using the anterior-posterior ankle position during this phase. Estimates of foot off and foot contact were calculated as detailed in the main text. Within this single-support phase, the samples representing missing data were identified and the percentage occlusion during the single-support phase was calculated. The distribution of occlusion over the single-support phase was visualized with a histogram presenting the percentage of all trials with occlusion during a specific part of the time-normalized single-support phase in bins of 5%.

The next step in the analysis was to introduce occlusion (i.e., based on observed occlusion for the right side) to the data of the typically unoccluded left side to examine the effect of occlusion (and the associated interpolation of missing data) on estimates of foot placement locations. This was done by using the observed occlusion during the right single-support phase of matched trials (i.e., in terms of distance and imposed step length). Subsequently, the so-obtained 'occluded' time series of the left ankle were interpolated with a spline algorithm before calculating foot placement locations and determining between-systems differences.

Statistical analysis

The amount of occlusion was assessed using a Distance (2m, 3m) by Side (left foot placement location, right foot placement location) repeated-measures ANOVA. The assumption of sphericity was checked according to Girden [1]. If Greenhouse–Geisser's epsilon exceeded 0.75, the Huynh–Feldt correction was applied; otherwise the Greenhouse–Geisser correction was used. Main effects were

examined with a LSD post hoc test. Paired-samples *t*-tests were used in case of a significant interaction. Effect sizes were quantified with η_p^2.

The between-systems differences for the foot placement locations of the left stepping trials were compared between original and 'occluded' data with a paired-samples *t*-test for each distance by imposed step length combination.

Results

The amount of occlusion differed significantly between distances (2m: 11.60±0.71%, 3m: 9.60±0.71%; $F(1,9)=6.41$, $p=0.032$, $\eta_p^2=0.416$) and sides (left: 0.07±0.07%, right: 21.13±1.16%; $F(1,9)=339.17$, $p<0.001$, $\eta_p^2=0.974$). Furthermore, there was a significant DistancexSide interaction ($F(1,9)=6.21$, $p=0.034$, $\eta_p^2=0.408$), revealing that the significant difference between the two distances was only evident for the right side with a significantly larger amount of occlusion for the 2m distance (2m: 23.11±4.40%, 3m: 19.15±4.48%; $t(9)=2.51$, $p=0.033$). In Figure 1, the amount and distribution of occlusion during the single-support phase in the left and right ankle data are depicted, presented separately for the two distances. As can be appreciated from the figure (right panel), occlusion in the single-support phase for the right ankle occurred earlier for the 2m distance than for the 3m distance, which may have contributed to the significant difference in the amount of occlusion between these two distances.

The original and 'occluded' data of the left ankle during the single-support phase are presented in Figure 2, separately for the 2m and 3m distance. The introduced missing data has little to no effect on the presented time series for both distances. This was confirmed by the results of the foot placement locations presented in Table 1. The bias in the between-systems differences of the foot placement locations calculated with the original and 'occluded' data were not present (i.e., identical values for the foot placement locations calculated with the original data and the 'occluded' data) or negligible (i.e., submillimeter biases with low amount of variation). These biases, if any, were not significant for both distances.

Conclusion

Occlusion in the Kinect v2 data cannot explain the more pronounced between-systems differences seen for foot placement locations and consequently step lengths for the right side at the 2m distance. Whereas the amount and timing of occlusion during the right single-support phase slightly differed between the 2m and 3m distance, the foot placement locations calculated with the 'occluded' data of the left ankle demonstrated negligible biases compared to the foot placement locations calculated with the original data, for both distances alike.

References

1. Girden, E.R. *ANOVA: Repeated measures*; Sage University Paper Series on Quantitative Applications in the Social Sciences: Newbury Park, United States, 1992.

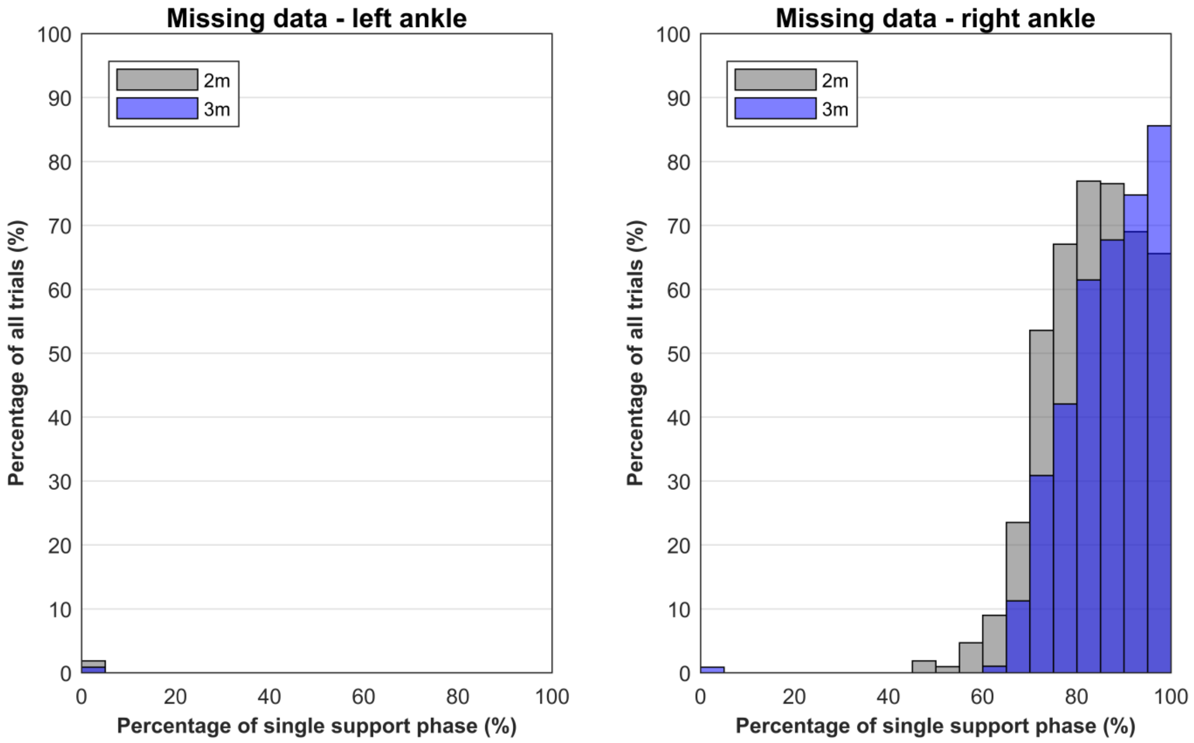

Figure 1. The amount and distribution of occlusion over the single-support phase, presented as the percentage of all trials with occlusion during a specific part of the time-normalized single-support phase in bins of 5%, for the left and right ankle (left and right panel, respectively), presented separately for the 2m (gray) and 3m (blue) distance.

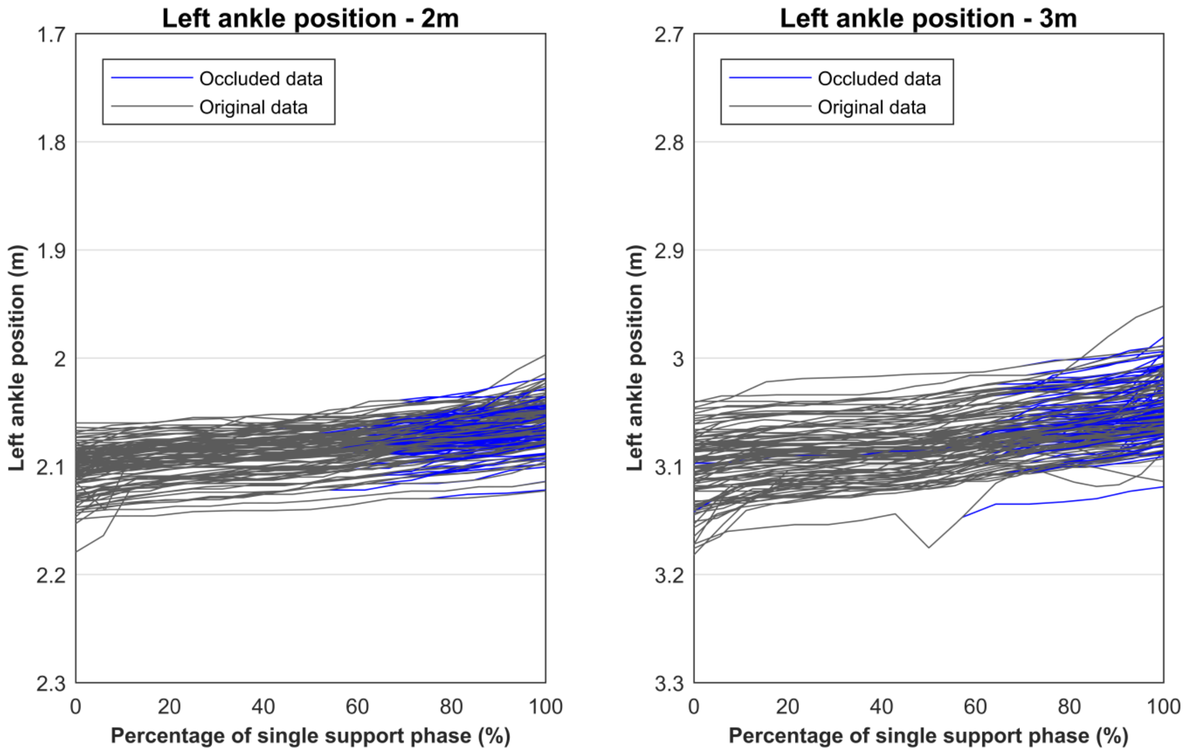

Figure 2. The original (gray) and 'occluded' (blue) time series of the left ankle in the anterior-posterior direction during the single-support phase, presented for the 2m and 3m distance (left and right panel, respectively).

Table 1. Mean values and between-subjects standard deviations (SD) of the between-systems differences (in cm) for foot placement locations calculated with the original and 'occluded' data of the left ankle, bias in between-systems differences and *t*-statistics.

			**Between-systems difference**				
			**Original**	**'Occluded'**	**Bias**		
			**Mean ± SD**	**Mean ± SD**	**Mean ± SD**	***t*(9)**	***p***
**Foot placement location (cm)**	**2m**	**50cm**	0.458 ± 1.220	0.464 ± 1.224	0.006 ± 0.019	1.00	0.343
		**60cm**	0.468 ± 1.290	0.468 ± 1.290	0 ± 0*	-	-
		**70cm**	0.625 ± 1.390	0.625 ± 1.390	0 ± 0*	-	-
		**80cm**	0.762 ± 1.326	0.762 ± 1.326	0 ± 0*	-	-
		**90cm**	0.747 ± 1.378	0.747 ± 1.378	0 ± 0*	-	-
	**3m**	**50cm**	0.579 ± 1.156	0.579 ± 1.156	0 ± 0*	-	-
		**60cm**	0.344 ± 1.392	0.344 ± 1.392	0 ± 0*	-	-
		**70cm**	0.699 ± 1.833	0.699 ± 1.833	0 ± 0*	-	-
		**80cm**	0.786 ± 1.944	0.801 ± 1.950	0.015 ± 0.046	1.00	0.343
		**90cm**	0.453 ± 1.723	0.405 ± 1.647	-0.048 ± 0.218	0.70	0.504

* Identical values for the foot placement locations calculated with the original and 'occluded' data of the Kinect v2 system.
